# A Three-Step, Single Session Therapy Intervention for COVID-Related Anxiety in a Pediatric Emergency Department

**DOI:** 10.7759/cureus.12371

**Published:** 2020-12-29

**Authors:** Diane P Lee, Scott A Simpson

**Affiliations:** 1 Psychiatry, University of Colorado Anschutz Medical Campus, Aurora, USA; 2 Psychiatric Emergency Services, Denver Health Medical Center, Denver, USA

**Keywords:** integrated behavioral health, pediatric emergency department, psychiatric emergency, covid-19

## Abstract

The novel Coronavirus disease (COVID-19) pandemic has led to increases in anxiety and depression, and mental health-related emergency department visits remain frequent despite overall changes in ED utilization. Here, we present a case of COVID-related anxiety and demonstrate the utility of a brief, single-session therapy intervention delivered in the ED. The growing mental health burden of COVID-19 suggests that pediatric health care providers will treat patients with COVID-related anxiety during this pandemic. This case demonstrates a common presentation of somatization of anxiety and outlines a three-step, cognitive-behavioral intervention that can be particularly effective in treating COVID-related anxiety in the context of a single ED or medical visit.

## Introduction

Before the novel coronavirus disease (COVID-19) pandemic, mental health visits to pediatric emergency departments were increasingly common. Over half a million children present to EDs for mental health reasons each year and EDs serve as the first point of contact for most children presenting with acute psychiatric issues [1]. Psychiatric visits are especially common among children from lower socioeconomic status backgrounds who have limited access to health care [2]. Furthermore, mental health symptoms are common among all children in the ED: almost half of the patients presenting to pediatric EDs meet the criteria for an anxiety disorder [3]. The COVID-19 pandemic has led to increases in anxiety and depression [4], and mental health-related ED visits remain frequent despite overall changes in ED utilization [5]. Children experience anxiety related to the epidemic and the health of relatives, poor sleep, physical discomfort, agitation, and separation anxiety [6].

Given that mental health symptoms often present initially as somatic complaints that require emergent medical assessment, pediatricians and pediatric health care providers will treat patients with COVID-related anxiety during this pandemic. Here, we present a case of one such presentation of somatization of anxiety in the ED and demonstrate the utility of brief single-session therapy intervention.

## Case presentation

A 10-year-old American Indian female was brought to the ED by her mother for chest pain, abdominal pain, nausea, and decreased appetite for the last several weeks. She had no significant medical or psychiatric history. Her vital signs and laboratory tests were reassuring. Upon initial assessment, the patient’s mother voiced concerns about the patient’s anxiety and distress related to COVID-19. The team offered the family consultation with the service’s integrated behavioral health consultant [7].

With the consultant, the patient’s mother reported that the COVID-19 pandemic had been difficult. The patient had been feeling “down” and experiencing increases in irritability, loss of pleasure (anhedonia), crying, racing thoughts, and worrying. The patient reported struggling with social isolation and being unable to see friends, as she was previously very active at school and with peers. Additionally, the patient worried about her family members contracting COVID; two of her siblings have chronic health conditions. She also worried that her abdominal pain was an indication of a serious health issue. The patient denied suicidal thoughts or substance use.

The behavioral health consultant began a brief cognitive-behavioral therapy (CBT) intervention. First, psychoeducation was provided on how common the patients’ concerns are amid the pandemic and how somatic complaints are a frequent manifestation of anxiety. Together, the patient, her mother, and the clinician identified the goals of improving the patient’s anxiety and finding ways for the patient to be more active despite physical distancing and quarantine restrictions. Coping skills and mindfulness training were provided so that the patient could manage episodes of anxiety and subsequent pain. During this teaching, the clinician identified cognitive distortions in the patient’s thinking and emphasized precautions that the family was taking to remain healthy-thereby challenging the patient’s catastrophic thinking that she needed to be doing more to protect her family. Additionally, the consultant reframed the patient’s thoughts that she was unable to engage in positive activities given COVID restrictions by reminding her that she still had many options for preferred activities still available to her. To encourage more frequent healthy behaviors, the family was encouraged to maintain routine in the day and facilitate social connections despite physical distancing.

The patient’s medical workup was reassuring, and other diagnoses or conditions unrelated to anxiety such as viral illness, appendicitis, and chest pain with cardiac etiology were assessed to be unlikely. At the time of ED discharge following this brief, CBT intervention, the patient reported significant decreases in anxiety level and somatic symptoms. Furthermore, the family felt reassured and motivated to reinforce the treatment plan discussed. In the six months following this visit, the patient had not returned to the ED.

## Discussion

Even before the COVID-19 pandemic, mental health presentations, including somatization of anxiety, were common in the ED. Furthermore, almost half of mental health-related visits to pediatric EDs are repeat visits, which reflects the lack of training, consensus, and protocols for evidence-based treatments for mental health symptoms in the ED setting [8]. This case reflects a common presentation of COVID-related anxiety which is more often better treated through brief intervention than through medication or even referrals to treatment. Additionally, this case provides guidance around the implementation of an evidence-based intervention. Single-session therapy can be particularly effective in reducing anxiety and is well-suited to be integrated into and delivered in the context of a medical visit by pediatric healthcare providers [9]. 

The general structure and content of this brief intervention are detailed in Table 1. Clinicians start with (1) providing teaching around the patient’s symptoms; this step not only teaches patients and families but also builds rapport before the following two steps. Next, the clinician (2) discusses cognitive and behavioral techniques for managing anxiety. Finally, (3) goals for aftercare are clearly identified. It is not uncommon for cognitive distortions-irrational thoughts that perpetuate anxiety-to arise during the assessment [10]. Table 2 lists common distortions and aids in challenging these distortions. Common cognitive distortions include catastrophization (assuming the worst possible outcome), fortune-telling (predicting negative outcomes), and black-and-white thinking (thinking exclusively in extremes). When using one of these reframing statements, the clinician should begin with a validating statement and then follow with a direct reframing of the patient’s cognitive distortion.

**Table 1:** A Three-Step, Brief CBT Intervention for Managing COVID-Related Anxiety CBT: cognitive-behavioral therapy

Step	Goal	Intervention	Potential Pitfalls
1. Psychoeducation	Provide validation and build rapport	Validate distress and anxiety	Consider referral for follow-up if symptoms persist beyond a single session.
	Assess if the patient is experiencing the effects of a discrete stressor (e.g., COVID) or the presence of underlying mental health symptoms which will require further follow up	Teach patients about the somatic manifestation of mental health symptoms	
		Teach about the general mental health burden of COVID	
2. Coping skills	Support healthier coping with anxiety	Teach mindfulness-based coping skills9 such as deep breathing, body scans, or progressive muscle relaxation	If the patient’s coping mechanisms include substance abuse or self-harming, further safety assessment is needed.
		Challenge thought distortions (Table 2)	
3. Behavioral activation	Increase the frequency of healthy and enjoyable behaviors	Set specific goals to engage in positive activities	Patients with complex social stressors (e.g., homelessness) are likely to need additional support and resources.
		Set goals related to maintaining a regular routine and social connection despite COVID restrictions	

**Table 2:** Common Thought Distortions Amid COVID and Responses

Patient Statement	Type of Distortion	Reframing Statement
“COVID will never end.”	Catastrophization	“I know it seems endless, but we have had pandemics before, and things got better. Many other places are already doing better.”
“I’m going to get my whole family sick”	Catastrophization	“I know you are worried about your family getting sick. Remember that you are doing a good job being careful and you are already doing everything you can to follow the safety guidelines.”
“My stomach hurts. There must be something seriously wrong with me.”	Catastrophization and somatization	“I know it’s scary when it feels like something is wrong with your body. When kids feel really worried, they often also feel pain in their stomach or chest. This does not mean that you are really sick or that you have COVID.”
“My parents will get COVID and die”	Catastrophization and fortune telling	“COVID is scary and you are really worried. Remember that your family is being careful to stay healthy and that doctors are working hard to take care of people who do get sick. Most people recover from COVID.”
“I can’t do anything fun because of COVID. I just have to do nothing.”	Black-and-white thinking	“It feels like you cannot do anything fun right now because of COVID and it is so hard to stay at home. Let’s think of what you can still do at home and how you can still spend time with friends even if you can’t see them.”

## Conclusions

Cognitive-behavioral therapy is a therapeutic philosophy that encourages behavior change as a way to reduce anxiety and depression, as one’s behaviors and emotions are inextricably linked. Furthermore, brief therapy is most helpful in the context of a discrete stressor as with COVID-19. This case demonstrates how pediatricians and pediatric health care providers can apply a brief, evidence-based CBT intervention, which is well-suited for presentations of COVID-related anxiety particularly in the context of a single medical visit. Additionally, this case reminds clinicians of the importance of providing quality mental health interventions for underserved patients who frequently present to the ED. Consistent with the effectiveness of evidence-based, single-session interventions, the patient presented in this case reported significant decreases in anxiety by the time of discharge. Furthermore, although almost half of mental health ED visits are repeat visits, this patient has not returned to the ED, which serves as another potential measure of effectiveness. 

Given the pandemic’s mental health impact on children and families, pediatricians and pediatric health care providers will treat patients with COVID-related anxiety. Furthermore, pediatricians’ and pediatric health care providers’ skills will be both challenged and needed in this time of heightened anxiety around COVID-19.
